# 固定化金属离子亲和发光二氧化硅纳米粒子的制备及其用于磷酸化蛋白免疫印迹标记

**DOI:** 10.3724/SP.J.1123.2020.05024

**Published:** 2021-04-08

**Authors:** Yuxiao MAO, Mengmeng ZHENG, Guizhen LIU, Baoli AN, Jingwu KANG

**Affiliations:** 1.上海大学理学院化学系, 上海 200444; 1. Department of Chemistry, College of Science, Shanghai University, Shanghai 200444, China; 2.中国科学院上海有机化学研究所, 生命有机化学国家重点实验室, 上海 200032; 2. State Key Laboratory of Bioorganic Chemistry and Natural Products Chemistry, Shanghai Institute of Organic Chemistry, Chinese Academy of Sciences, Shanghai 200032, China

**Keywords:** 免疫印迹, 固定化金属离子亲和, 发光二氧化硅纳米粒子, 磷酸化蛋白标记, Western Blot, immobilized metal ion affinity, luminescent silica nanoparticles, phosphorylated protein labelling

## Abstract

发展了一种能够识别磷酸化蛋白的固定化金属离子亲和发光二氧化硅纳米粒子用于免疫印迹(Western Blot)磷酸化蛋白的标记。首先通过反相微乳液Stöber方法合成了掺杂异硫氰酸荧光素硅烷化衍生物的发光二氧化硅(FITC@SiO_2_)球形纳米粒子,粒子平均粒径为60 nm。然后通过共聚反应在FITC@SiO_2_纳米粒子表面生成一层聚合物用于保护纳米粒子,并引入*N*,*N*-(双羧甲基)-L-赖氨酸功能基团用于螯合金属离子,从而实现固定化金属离子亲和作用。以*α*-酪蛋白作为实验模型,用高效液相色谱-质谱研究了螯合不同金属离子的发光纳米粒子对磷酸化蛋白的识别作用。结果表明,螯合了Ti^4+^金属离子的发光二氧化硅FITC@SiO_2_纳米粒子对*α*-酪蛋白酶解液中的磷酸化肽段的富集作用最强。利用这种发光二氧化硅FITC@SiO_2_纳米粒子对磷酸化肽段的特异性识别性能,可用于Western Blot实验中标记磷酸化蛋白的条带。结果显示,*α*-酪蛋白的电泳条带可以被亲和发光二氧化硅FITC@SiO_2_纳米粒子标记,而作为对照的牛血清白蛋白则没有被标记。

## Abstract (en)

Protein phosphorylation is an important type of post-translational protein modification. In Western Blot experiment, the assay of phosphoproteins need special phospho antibodies, which are expensive, difficult to preserve, poorly reproducible. To this end, the immobilized metal ion affinity luminescent silica nanoparticles for instead of phospho antibodies were prepared. A layer of polymer was created on the surface of the silica nanoparticles via co-polymerization to protect the nanoparticles and to functionalize them with the immobilized metal ion affinity property to specifically label the phosphorylated proteins in Western Blot assays. The affinity luminescent silica nanoparticles were prepared with the following procedure. First, the sol-gel precursor fluorescein isothiocyanate-3-aminopropyltriethoxysilane (FITC-APTES) with the fluorescent moiety was prepared by modifying APTES with FITC. The luminescent silica nanoparticles (FITC@SiO_2_) were synthesized using the Stöber synthesis method in a reversed microemulsion. Briefly, 123.2 mL of cyclohexane, 25.6 mL of *n*-hexanol, and 5.44 mL of deionized water were ultrasonically mixed, and then 28.3 g of Triton X-100 were added and the mixture was magnetically stirred for 15 min to form a clear and transparent microemulsion system. Within 10 min, 0.8 mL of FITC-APTES precursor, 1.6 mL of tetraethoxysilane (TEOS), and 0.96 mL of concentrated ammonia (25%-27%, mass fraction) were added to the microemulsion, and the mixture was stirred at 24 ℃ for 24 h. After the reaction, the microemulsion system was destroyed by adding 200 mL of ethanol. The resulting FITC@SiO_2_ luminescent silica nanoparticles were centrifuged, and washed three times with ethanol. After dryness, the FITC@SiO_2_ nanoparticles were modified with methacryloxy-propyltrimethoxysilane (MPS) to introduce the double bonds for further modification. The functional monomer nitrilotriacetic acid (NTA) and glycidyl methacrylate (GMA) were copolymerized on the surface of the nanoparticles to convert FITC@SiO_2_-MPS to FITC@SiO_2_-MPS-GMA-NTA. The polymer coating of the silica nanoparticles was not only able to protect the silica from hydrolysis, but also to introduce the functional groups of nitrilotriacetic acid, which can chelate with metal ions. Elemental analysis demonstrated that the NTA groups had been bonded to the surface of the nanoparticles via copolymerization. The polymerization did not affect the morphology and fluorescence properties of the nanoparticles. The FITC@SiO_2_-MPS-GMA-NTA nanoparticles were activated with three different metal ions Zr^4+^, Fe^3+^, and Ti^4+^, for the enrichment of phosphorylated peptides derived form *α*-casein tryptic digestion. HPLC-MS analysis indicated that the FITC@SiO_2_-MPS-GMA-NTA-Ti ^4+^ nanoparticles are the best for the enrichment of phosphorylated peptides. The FITC@SiO_2_-MPS-GMA-NTA-Ti^4+^ nanoparticles were used for labelling the phosphorylated proteins in Western Blot experiment. The electrophoretic band of *α*-casein could be clearly labeled with the FITC@SiO_2_-MPS-GMA-NTA-Ti ^4+^ nanoparticles, while the bovine albumin band could not be labelled. This indicates that the luminescent FITC@SiO_2_-MPS-GMA-NTA-Ti^4+^nanoparticles can be used to label the phosphorylated proteins in Western Blot experiments.

蛋白质磷酸化是生命过程中调节和控制蛋白质活力和功能的最基本、最普遍、最重要的机制^[1,2]^。在生物体中只有大约1%~2%的蛋白质会发生磷酸化修饰,在质谱检测中会受到大量非磷酸化肽段的干扰^[3]^。发展富集磷酸化肽段的技术是提高磷酸化蛋白分析的重要实验方法^[4]^。免疫沉淀法^[5,6]^、固定金属亲和色谱法^[7,8]^和金属氧化物亲和色谱法^[9,10]^是磷酸化蛋白分离和富集最为常见的方法。由于不同特性的纳米复合材料能够选择性结合磷酸化多肽和磷酸化蛋白,因此纳米复合材料常用于磷酸化蛋白的分离与鉴定^[11,12]^。将磷酸化蛋白的分离与富集技术和生物质谱技术联合使用是分析磷酸化蛋白的常用技术。磁性纳米粒子经常用于富集磷酸化蛋白和磷酸化肽段^[13,14,15,16]^。

免疫印迹(Western Blot)是分子生物学和生物化学中常用的一种实验方法,其基本原理是将电泳分离后的细胞或组织总蛋白质从凝胶转移到固相支持物硝酸纤维素膜或聚偏二氟乙烯膜上,然后用特异性抗体检测某特定蛋白质的检测技术。采用免疫印迹分离和鉴定磷酸化蛋白时,需要借助磷酸化抗体对磷酸化蛋白进行特异性识别,且需使用二抗放大检测信号^[17]^。但是,磷酸化抗体价格昂贵,难以保存,而且不同批次之间的重现性不好。因此,亟须发展一种制备过程简单、容易保存、成本低廉、对磷酸化蛋白具有特异性识别的纳米发光材料代替磷酸化抗体。发光二氧化硅纳米粒子常用于细胞成像分析^[18,19,20]^,目前未发现将发光二氧化硅纳米粒子用于测定磷酸化蛋白的文献报道。

本工作合成了一种包覆异硫氰酸荧光素的发光二氧化硅纳米粒子FITC@SiO_2_,并将其用于磷酸化蛋白的富集和荧光识别分析。将能够富集和识别磷酸基团的用于螯合金属离子Ti^4+^的*N*,*N*-(双羧甲基)-L-赖氨酸的连接臂通过共聚反应键合到FITC@SiO_2_纳米粒子表面上。修饰后的FITC@SiO_2_纳米粒子可以直接富集和荧光识别磷酸化蛋白。FITC@SiO_2_纳米粒子带有荧光,标记凝胶电泳条上的磷酸化蛋白条带后,使用凝胶成像系统可以清楚地观测到磷酸化蛋白被标记后的荧光信号。这是首次将发光二氧化硅FITC@SiO_2_纳米粒子与蛋白免疫印迹法结合,本实验方法可以大大降低磷酸化蛋白的检测成本。发光二氧化硅FITC@SiO_2_纳米粒子还具有较稳定的化学性质,有望显著提高磷酸化蛋白的检测重现性。

## 1 实验部分

### 1.1 仪器与试剂

荧光光谱仪(FLS1000, Edinburgh Instruments,英国); Ultimate 3000 HPLC-LTQ XL质谱联用仪(赛默飞世尔,美国)。Bio-Rad GelDoc EZ凝胶成像系统(Bio-Rad,美国)。

过硫酸铵(APS)、四乙氧基硅烷(TEOS)、荧光素异硫氰酸酯(FITC)、3-氨丙基三乙氧基硅烷(APTES)、硫酸钛(Ti(SO_4_)_2_)、六水合氯化铁(FeCl_3_·6H_2_O)、八水合氯氧化锆(ZrOCl_2_·8H_2_O)、甲基丙烯酸缩水甘油酯(GMA)、*N*,*N*,*N'*,*N*'-四甲基乙二胺(TEMED)、三羟甲基氨基甲烷(Tris)、甘氨酸、Triton-X100、丙烯酰胺、三氟乙酸(TFA)和甲酸(HCOOH)购自Fluka公司(美国);正己醇和环己烷购自上海化学试剂有限公司;甲基丙烯酰氧丙基三甲氧基硅烷(MPS)、3-(三羟丙基)甲基膦酸丙酯(THMPS)、预染蛋白标准品、*α*-酪蛋白和胰蛋白酶购自Sigma-Aldrich公司(美国);戊烯酸、*N*-羟基琥珀酰亚胺和1-乙基-(3-二甲基氨基丙基)碳酰二亚胺盐酸盐(EDC·HCl)购自上海安耐吉化学公司;氨水、*N*,*N*-二甲基甲酰胺(DMF)、二氯甲烷(CH_2_Cl_2_)、盐酸(35%~37%,分析纯)、碳酸氢钠和4-二甲氨基吡啶(DMAP)购自梯希爱(上海)公司;*N*,*N*-(双羧甲基)-L-赖氨酸购自毕得医药公司(上海); *β*-巯基乙醇购自Amresco公司(美国);牛血清白蛋白(BSA)、碳酸氢铵和氯化钠均购自国药集团(北京);乙腈(ACN)和甲醇购自Merck公司(德国)。实验用水均经过Millipore净水器处理。

### 1.2 FITC@SiO_2_纳米粒子的合成及其表面功能化修饰

FITC-APTES前驱体的合成:在避光条件下,向塑料瓶中依次加入10.5 mg FITC、2 mL无水乙醇和138 μL APTES,磁力搅拌,反应12 h后得到FITC-APTES前驱体。使用反相微乳液法Stöber方法合成发光二氧化硅FITC@SiO_2_纳米粒子^[21]^。将123.2 mL环己烷、25.6 mL正己醇和5.44 mL去离子水超声混合,然后加入28.3 g Triton X-100,磁力搅拌15 min,形成澄清透明的微乳液体系。在10 min内依次向微乳液中加入0.8 mL FITC-APTES前驱体、1.6 mL TEOS和0.96 mL浓氨水(25%~27%,质量分数),于24 ℃下搅拌24 h。反应结束后,用200 mL无水乙醇破坏微乳液体系,离心分离,用无水乙醇超声洗涤3次,得到发光二氧化硅FITC@SiO_2_纳米粒子。

FITC@SiO_2_-MPS发光二氧化硅纳米粒子的合成:在一个干燥的250 mL广口瓶中,将FITC@SiO_2_纳米粒子分散于100 mL干燥的无水乙醇中,超声15 min,使FITC@SiO_2_纳米粒子充分分散。在体系中加入1 mL MPS,于35 ℃油浴中反应4 h。反应完毕后,离心分离,用无水乙醇超声洗涤3次,即得FITC@SiO_2_-MPS纳米粒子。

功能单体2-(双羧甲基氨基)-6-戊-4-烯酰氨基己酸(NTA)^[22]^的合成:在一个干净的250 mL烧瓶中,加入4.5 g *N*-羟基琥珀酰亚胺,抽换氮气3次后,加入150 mL二氯甲烷,搅拌均匀后,加入3 mL戊烯酸,待白色粒子分散,溶液呈澄清透明状后,加入6.9 g EDC·HCl和4.0 g DMAP,室温搅拌12 h,旋转蒸发浓缩后,产物进行柱层析分离,旋转蒸发浓缩后得到白色晶体4-戊烯酸琥珀酰亚胺酯5.13 g,产率为86.9%。将一定量的4-戊烯酸琥珀酰亚胺酯溶于水中,制成质量浓度为200 mg/mL的溶液,用NaHCO_3_调至pH 8后,取5 mL 4-戊烯酸琥珀酰亚胺酯的水溶液,加入2.5 g的*N*,*N*-双羧甲基-L-赖氨酸,溶解后室温搅拌2 h,产物进行柱层析分离,旋转蒸发后得到1.53 g 2-(双羧甲基氨基)-6-戊-4-烯酰氨基己酸,产率为88.9%。

带有三羧酸基团的FITC@SiO_2_-MPS-GMA-NTA发光二氧化硅纳米粒子的合成:在一个干净的50 mL圆底烧瓶中,加入30 mg FITC@SiO_2_-MPS中间体、20 mL去离子水,超声30 min分散,加入1 mL无水乙醇,超声10 min,加入0.2 mL GMA、10 mg NTA功能单体。在氮气保护下搅拌加热。当温度升至80 ℃时,用注射器慢慢加入0.1 mL新配的10%过硫酸铵水溶液,搅拌,反应24 h,然后以8300 r/min离心分离1 min,用乙醇超声洗涤粒子3次,得到带有三羧酸结构的FITC@SiO_2_-MPS-GMA-NTA纳米粒子。合成的反应流程如图1所示。

**图 1:**
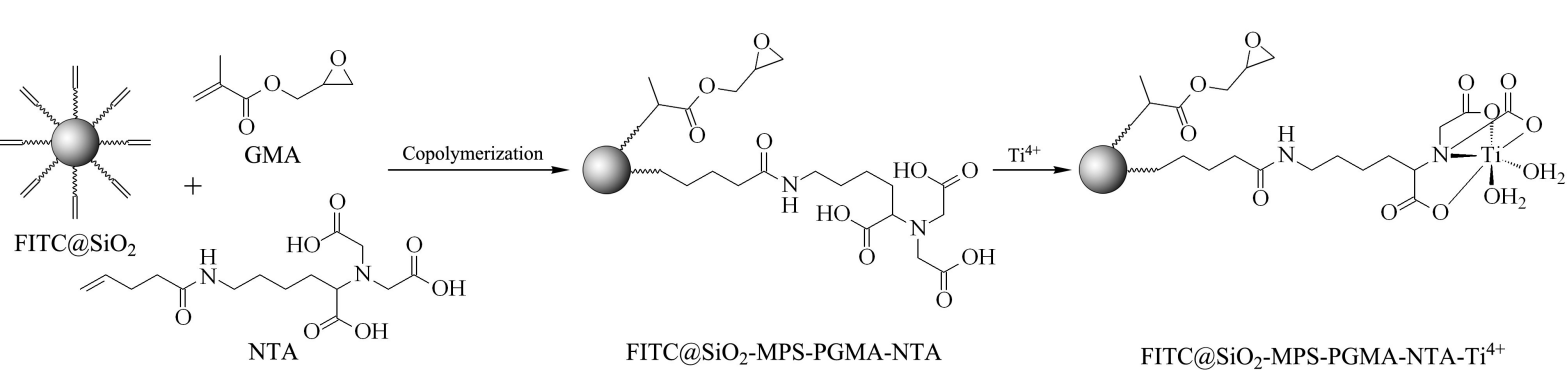
FITC@SiO_2_-MPS-GMA-NTA-Ti^4+^发光二氧化硅纳米粒子的合成流程示意图

分别配制100 mg/mL硫酸钛、氯化锆、氯化铁水溶液,将0.10 g FITC@SiO_2_-MPS-GMA-NTA纳米粒子分别加入上述不同离子的水溶液中。在室温下孵育1 h后,连接臂上的三羧酸基团可以成功地螯合金属离子。

将纳米粒子离心分离,用超纯水洗涤3次,除去未螯合的金属离子,真空干燥后即可得螯合不同金属离子的发光二氧化硅纳米粒子(FITC@SiO_2_-MPS-GMA-NTA-Zr^4+^、FITC@SiO_2_-MPS-GMA-NTA-Zr^4+^、FITC@SiO_2_-MPS-GMA-NTA-Fe^3+^),并于4 ℃保存,待用。

### 1.3 磷酸化肽段和磷酸化蛋白的预处理

FITC@SiO_2_-MPS-PGMA-NTA-Ti^4+^纳米粒子用于磷酸化肽段的富集。分别称取1 mg牛血清白蛋白、*α*-酪蛋白于Eppendorf管中,加入胰蛋白酶过夜酶解。同时配制缓冲液A(200 mmol/L NaCl水溶液(含有50%ACN和6% TFA)、缓冲液B(30%乙腈水溶液(含0.1% TFA))、缓冲液C(80%乙腈水溶液(含/6% TFA))、蛋白质洗脱液(10%氨水溶液)。先用缓冲液A洗涤FITC@SiO_2_-MPS-GMA-NTA-Ti^4+^粒子,洗涤3次后,将0.1 mg FITC@SiO_2_-MPS-GMA-NTA-Ti^4+^粒子分散于1 mL缓冲液A中。将酶解后的蛋白质溶液(1 mg/mL)与缓冲液C按体积比1:1混合,孵育1 h。在FITC@SiO_2_-MPS-GMA-NTA-Ti^4+^充分吸附肽段后,以10000 r/min离心分离1 min,弃去上清液。用缓冲液A和B分别洗涤FITC@SiO_2_-MPS-GMA-NTA-Ti^4+^粒子,以除去纳米粒子上残留的非磷酸化肽段。最后使用蛋白质洗脱液洗脱FITC@SiO_2_-MPS-GMA-NTA-Ti^4+^粒子上吸附的磷酸化肽段,收集洗脱液,冷冻干燥,溶于含2%乙腈和0.1%TFA的水溶液中,最后进行HPLC-MS分析。

### 1.4 分析条件

色谱柱:Agilent ZORBAX SB-C18柱(250 mm×4.6 mm, 5 μm);柱温:25 ℃;流动相A:水(含0.1%甲酸);流动相B:乙腈;流速:1 mL/min。洗脱梯度程序:0~25 min, 5%B~45%B; 25~30 min, 45%B~75%B; 30~35 min, 75%B; 35~40 min, 75%B~5%B; 40~50 min, 5%B。进样体积:10 μL。

HPLC与LTQ-Fleet离子阱质谱联用,在正离子模式下进行所有质谱信号的采集;喷雾电压为4.0 kV;鞘气流速为40 L/min;辅助气流速为10 L/min;金属毛细管温度为320 ℃;归一化的碰撞能量为35%;质量扫描范围为*m/z* 300~2000。

### 1.5 发光二氧化硅纳米粒子标记磷酸化蛋白

FITC@SiO_2_-MPS-GMA-NTA-Ti^4+^纳米粒子用于标记磷酸化蛋白。配制0.1 mg/mL的*α*-酪蛋白和牛血清白蛋白样品进行免疫印迹电泳。电泳所用胶条由15%(质量分数)分离胶和5%(质量分数)浓缩胶组成。每个泳道上样15 μL, 80 V电压堆积10 min后加压至110 V,约1 h后停止电泳。电泳完成后,在100 V电压下将胶条上的蛋白条带转移到硝酸纤维素膜上,转膜时间约为1 h。

称取5.8 g Tris、2.9 g甘氨酸、0.37 g十二烷基硫酸钠(SDS)和1 mL Tween-20,加入200 mL甲醇后定容至1 L容量瓶中,配制得到的溶液称为TBST溶液。用TBST溶液洗去硝酸纤维素膜上的转膜液,同时将硝酸纤维素膜浸泡在含有FITC@SiO_2_-MPS-GMA-NTA-Ti^4+^纳米粒子的TBST溶液(0.1 mg/mL)中,常温下摇荡30 min,使蛋白质与纳米粒子充分结合。随后使用TBST溶液浸泡硝酸纤维素膜,摇荡30 min以去除膜上未能与磷酸化蛋白结合的FITC@SiO_2_-MPS-GMA-NTA-Ti^4+^纳米粒子。该过程重复3次。洗涤结束后,将硝酸纤维素膜置于Bio-Rad GelDoc EZ凝胶成像系统下扫描成像,得到经FITC@SiO_2_-MPS-GMA-NTA-Ti^4+^纳米粒子染色后的磷酸化蛋白的荧光图像。

## 2 结果与讨论

### 2.1 FITC@SiO_2_纳米粒子的制备和表征

通过反相微乳液体系合成的FITC@SiO_2_纳米粒子具有光滑的球形外形。本文首先优化了前驱体中FITC与APTES的物质的量之比,制备了6种FITC@SiO_2_纳米粒子。将FITC@SiO_2_纳米粒子分别分散在无水乙醇中,配制成质量浓度为0.1 mg/mL的待测溶液,设定激发波长为488 nm,分别测定其发射光谱,最大发射波长为526 nm。测定结果如图2所示,随着APTES含量逐渐升高,FITC@SiO_2_纳米粒子的荧光强度也逐渐增强,当FITC: APTES物质的量比例为1:20时,荧光强度达到最大值。随后再增加APTES的含量,FITC@SiO_2_纳米粒子的荧光强度反而减小。因为当FITC的含量较多,而APTES不足时,无法形成足够的FITC-APTES前驱体。在TEOS的水解过程中,游离的FITC不能被包覆在SiO_2_纳米粒子中。因此选用FITC:APTES=1:20(物质的量)作为制备前驱体的比例。

**图 2:**
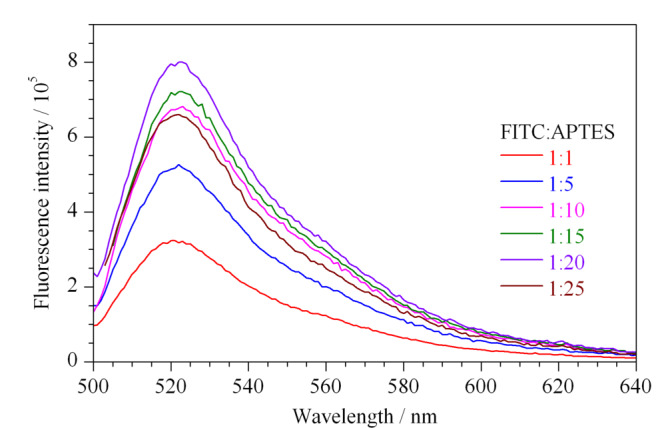
以不同前驱体(FITC和APTES)物质的量之比合成的发光二氧化硅FITC@SiO_2_纳米粒子的荧光发射光谱图

为了确认聚合物膜是否包裹在FITC@SiO_2_纳米粒子表面,我们对FITC@SiO_2_纳米粒子进行了元素分析。FITC@SiO_2_纳米粒子的碳、氮含量均小于0.3%,氢含量为0.5%,经过MPS修饰后的FITC@SiO_2_-MPS粒子的碳含量为0.76%,氮含量小于0.3%,氢含量为0.44%,说明有少量的MPS修饰在FITC@SiO_2_纳米粒子表面。与FITC@SiO_2_纳米粒子相比,经过表面共聚后现成的纳米粒子FITC@SiO_2_-MPS-GMA-NTA的碳、氮含量均大幅上升,碳含量为4.98%,氮含量为0.91%,结果说明NTA基团已经与GMA共聚在纳米粒子表面。

对FITC@SiO_2_、FITC@SiO_2_-MPS和FITC@SiO_2_-MPS-GMA-NTA纳米粒子分别进行透射电镜表征。图3a和图3b分别为FITC@SiO_2_和FITC@SiO_2_-MPS的TEM图,通过反相微乳液体系合成的FITC@SiO_2_纳米粒子具有光滑的球形外形,粒径分布比较均匀,FITC@SiO_2_和FITC@SiO_2_-MPS的平均粒径为60 nm。图3c对应的是FITC@SiO_2_-MPS-GMA-NTA纳米粒子的TEM图,可以看出,FITC@SiO_2_-MPS粒子表面形成一层聚合物涂层GMA和NTA基团后,对FITC@SiO_2_粒子的形貌影响很小,仍是表面光滑的球形粒子,FITC@SiO_2_-MPS-GMA-NTA的整体粒径没有发生明显的变化,直径仍大约为60 nm。

**图 3:**
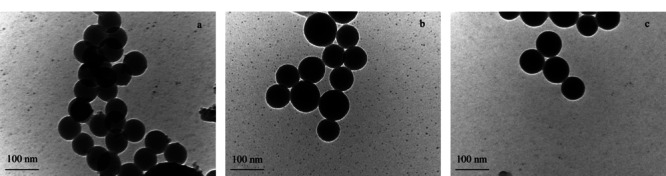
FITC@SiO_2_纳米粒子的TEM照片

将不同质量浓度(10^-5^~1 mg/mL)的FITC@SiO_2_-MPS-GMA-NTA纳米粒子分散在水中,在365 nm紫外灯照射下,不同浓度的粒子均发出绿色荧光,荧光的强度由弱到强(见图4)。各粒子的水分散性均较好,静置1 d后均无明显的沉降现象。

**图 4:**
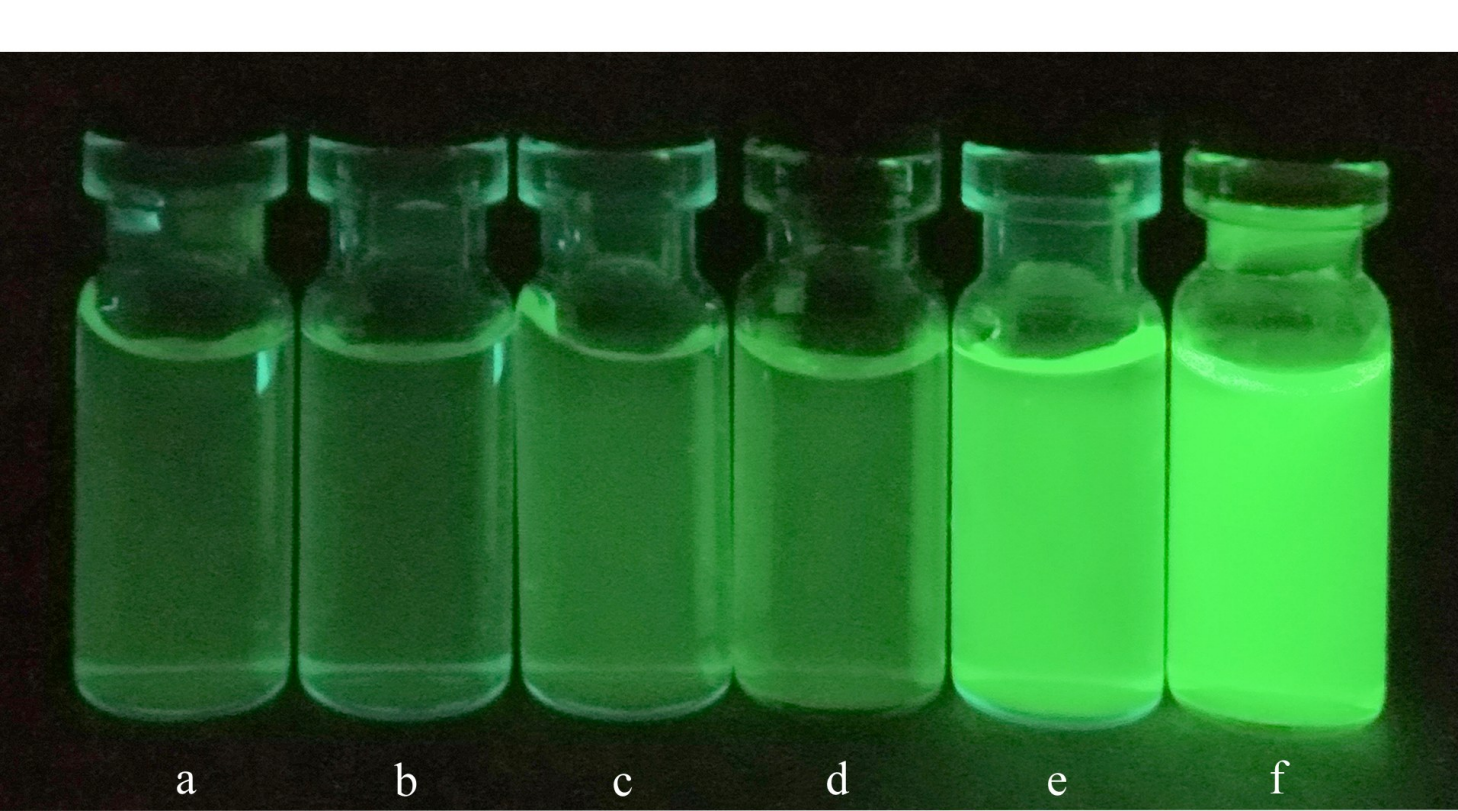
不同浓度FITC@SiO_2_-MPS-GMA-NTA在365 nm紫外灯照射下的荧光成像

### 2.2 发光二氧化硅纳米粒子对磷酸化肽段和磷酸化蛋白的特异性识别

实验选择了3种金属离子与发光二氧化硅纳米粒子形成固定化金属离子亲和配体(FITC@SiO_2_-MPS-GMA-NTA-Fe^3+^、FITC@SiO_2_-MPS-GMA-NTA-Zr^4+^和FITC@SiO_2_-MPS-GMA-NTA-Ti^4+^),并分别富集*α*-酪蛋白酶解液中的磷酸化肽段,色谱分析结果见图5。FITC@SiO_2_-MPS-GMA-NTA-Ti^4+^可以从*α*-酪蛋白酶解液中富集到10条磷酸化肽段(见图5a)。色谱图中的非磷酸化肽段的信号峰很低,谱图比较干净,说明FITC@SiO_2_-MPS-GMA-NTA-Ti^4+^纳米粒子能够特异性地富集和识别磷酸化肽段。如图5b所示,FITC@SiO_2_-MPS-GMA-NTA-Zr^4+^纳米粒子富集到7条磷酸化肽段,磷酸化肽段的色谱峰信号较强,并且谱图也较干净,说明FITC@SiO_2_-MPS-GMA-NTA-Zr^4+^粒子的非特异性吸附较少,但是Zr^4+^作为识别基团的纳米粒子富集后谱图的色谱峰信号强度约为Ti^4+^的50%。如图5c所示,FITC@SiO_2_-MPS-GMA-NTA-Fe^3+^纳米粒子从*α*-酪蛋白酶解液中富集到了4条磷酸化肽段,表明FITC@SiO_2_-MPS-GMA-NTA-Fe^3+^纳米粒子富集和识别磷酸化肽段的效果最差。实验结果表明,FITC@SiO_2_-MPS-GMA-NTA-Ti^4+^纳米粒子富集和识别磷酸化肽段的性能最好。表明用聚合物GMA包裹FITC@SiO_2_纳米粒子以减少非特异性吸附肽段的实验结果是成功的。

**图 5:**
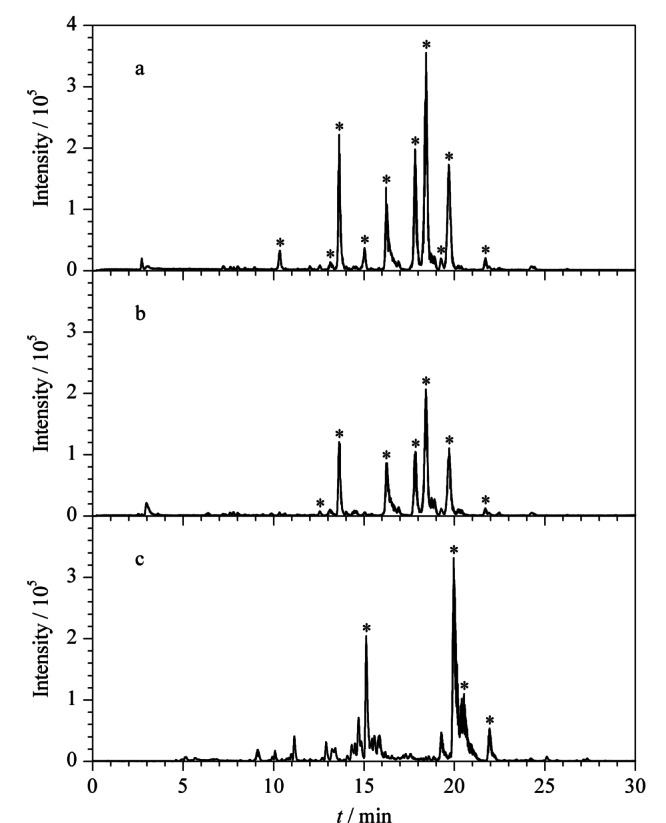
不同纳米粒子富集*α*-酪蛋白标准酶解液中的磷酸化肽段的色谱图

**图 6:**
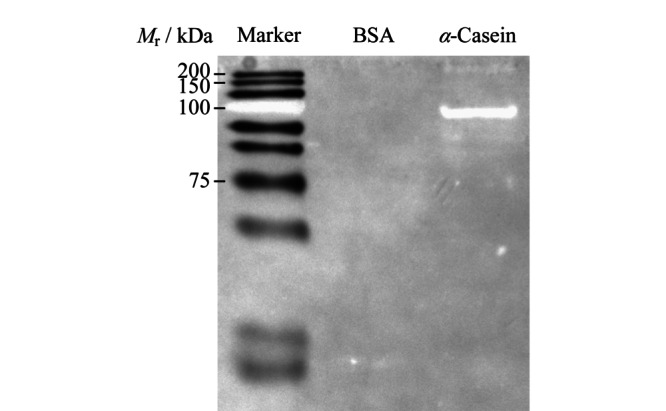
经过FITC@SiO_2_-MPS-GMA-NTA-Ti^4+^标记的*α*-酪蛋白免疫印迹电泳图

将*α*-酪蛋白和BSA分别在2条电泳泳道内进行聚丙烯酰胺凝胶电泳(SDS-PAGE)分离后,将得到的蛋白质条带转移到硝酸纤维素膜上,用合成的FITC@SiO_2_-MPS-GMA-NTA-Ti^4+^纳米粒子对其进行荧光染色。由图6可以看出,*α*-酪蛋白被成功染色,在荧光灯照射下发出荧光,由于BSA中不含有磷酸化蛋白,BSA蛋白条带的位置没有检测到荧光信号。而标记蛋白条带的100 kDa处也是磷酸化蛋白,也产生了荧光信号。实验结果表明,合成的FITC@SiO_2_-MPS-GMA-NTA-Ti^4+^纳米粒子能够特异性识别磷酸化蛋白,且不会与其他种类的蛋白质发生非特异性吸附。合成的FITC@SiO_2_-MPS-GMA-NTA-Ti^4+^纳米粒子同时起到识别磷酸化蛋白和放大信号的作用,并且其化学性质稳定,易于保存。表明FITC@SiO_2_-MPS-GMA-NTA-Ti^4+^纳米粒子可以用于Western Blot实验中代替磷酸化抗体。

## 3 结论

本文成功合成了单分散球形的可以螯合金属离子的发光二氧化硅纳米粒子FITC@SiO_2_-MPS-GMA-NTA-Ti^4+^,用以富集和识别磷酸化的蛋白质。在Western Blot分析磷酸化蛋白时,使用FITC@SiO_2_-MPS-GMA -NTA-Ti^4+^纳米粒子可以特异性荧光标记磷酸化蛋白,产生明亮的荧光条带,而对非磷酸化蛋白不发生标记作用。FITC@SiO_2_-MPS-GMA-NTA-Ti^4+^纳米粒子与磷酸化蛋白的特异性好,荧光强度高,可以在室温下保存较长时间,因此可广泛用于磷酸化蛋白的电泳成像。
